# Tackling Adolescent Substance Abuse in Hong Kong: Where We Should and Should Not Go

**DOI:** 10.1100/tsw.2007.315

**Published:** 2007-12-19

**Authors:** Daniel T. L. Shek

**Affiliations:** ^1^ Centre for Quality of Life, Hong Kong Institute of Asia-Pacific Studies, The Chinese University of Hong Kong, Hong Kong; ^2^ Kiang Wu Nursing College of Macau, Macau; ^3^ Social Welfare Practice and Research Centre, The Chinese University of Hong Kong, Hong Kong

**Keywords:** substance abuse, risk factors, Chinese, Hong Kong

## Abstract

In the 2007 Policy Address, the Chief Executive of the Hong Kong Special Administrative Region, P.R.C. expressed the Administration’s concern about adolescent substance abuse and proposed to form a high-level interdepartmental task force to tackle the problem in a holistic manner. In this paper, the author presents his observations about adolescent substance abuse in Hong Kong, and outlines the risk factors and related strategies based on the ecological perspective that the Government should consider in order to tackle the problem of adolescent substance abuse in Hong Kong. Furthermore, the directions where the Government should and should not go are discussed.

## INTRODUCTION

A survey of the websites of several international organizations (e.g., Office on Drugs and Crime of the United Nations, International Narcotics Control Board, National Institute of Drug Abuse in the United States, and European Monitoring Center for Drugs and Drug Addiction) shows that illicit drug use is a thorny global problem to be resolved. Probably because of the influence of popular culture and youth subculture, substance abuse among young people has also become an acute global problem. As Hong Kong is an international city where information flow (including those related to psychotropic drugs) is very quick, adolescent substance abuse is also a grave concern for Hong Kong[1,2].

An examination of the substance abuse figures in the past 20 years showed that there were two peaks in the substance abusers figures reported to the Central Registry of Drug Abuse (CRDA) maintained by the Narcotics Division of the Government. The first peak was in the mid-1990s, which was mainly related to easy access to tranquilizers not tightly controlled by legislations. The second peak was in the early 2000s, which was closely related to the rave party culture. In fact, these peaks mirrored the global trend of abusing nonopiate psychotropic substances, and the growing belief among young people that psychotropic substance abuse is nonaddictive and a valid choice of life[3].

Judging from the figures reported to the CRDA in recent years (see Table 1), it is clear that the present moment is not the worst time for Hong Kong in terms of adolescent substance abuse. In fact, this situation can be attributed to the multiprolonged approach adopted by the Narcotics Division and the

**TABLE 1**
**Substance Abuse Figures Reported in the Central Registry of Drug Abuse**

| Year | 1997 | 1998 | 1999 | 2000 | 2001 | 2002 | 2003 | 2004 | 2005 | 2006 |
|---|---|---|---|---|---|---|---|---|---|---|
| All drug abusers | 17,635 | 16,992 | 16,314 | 18,335 | 18,513 | 17,966 | 15,790 | 14,854 | 14,113 | 13,204 |
| *Mean age* | *34* | *34* | *35* | *32* | *33* | *34* | *34* | *35* | *35* | *34* |
| Male | 15,398 | 14,838 | 14,147 | 15,355 | 15,640 | 14,780 | 13,272 | 12,200 | 11,448 | 10,670 |
| *% of all* | *87.3* | *87.3* | *86.7* | *83.7* | *84.5* | *82.3* | *84.1* | *82.1* | *81.1* | *80.8* |
| *Mean age* | *35* | *35* | *36* | *34* | *34* | *35* | *36* | *36* | *37* | *36* |
| Female | 2,237 | 2,154 | 2,167 | 2,980 | 2,873 | 3,186 | 2,518 | 2,654 | 2,665 | 2,534 |
| *% of all* | *12.7* | *12.7* | *13.3* | *16.3* | *15.5* | *17.7* | *15.9* | *17.9* | *18.9* | *19.2* |
| *Mean age* | *27* | *27* | *28* | *25* | *26* | *27* | *28* | *28* | *28* | *27* |
| Young persons aged under 21 | 3,150 | 2,841 | 2,482 | 4,020 | 3,902 | 3,002 | 2,207 | 2,186 | 2,276 | 2,549 |
| *% of all* | *17.9* | *16.7* | *15.2* | *21.9* | *21.1* | *16.7* | *14.0* | *14.7* | *16.1* | *19.3* |
| *Mean age* | *18* | *18* | *18* | *17* | *17* | *17* | *17* | *17* | *17* | *17* |
| Newly reported persons | 3,614 | 3,417 | 3,135 | 5,395 | 5,644 | 5,241 | 4,444 | 3,760 | 3,723 | 3,482 |
| *% of all* | *20.5* | *20.1* | *19.2* | *29.4* | *30.5* | *29.2* | *28.1* | *25.3* | *26.4* | *26.4* |
| *Mean age* | *24* | *24* | *25* | *23* | *23* | *24* | *25* | *24* | *23* | *23* |

Action Committee against Narcotics of the Government of the Hong Kong Special Administrative Region, P.R.C. in the past years. Nevertheless, although figures on adolescent substance abusers reported to the CRDA presented in Table 1 appeared to stabilize and decline in recent years, there are several observations that deserve our attention. First, as shown in Table 2, drugs abused by young people under the age of 21 were mainly psychotropic substances, particularly ketamine. Actually, ketamine abuse in Hong Kong could be regarded as quite unique because this drug is not commonly abused in other parts of the world. Second, with the return of Hong Kong to China in 1997, traveling between Hong Kong and Shenzhen in mainland China has become very popular, hence creating the problem of cross-border adolescent substance abuse. Actually, with the use of electronic home-return permits, adolescents can easily go back to Shenzhen to abuse drugs without leaving any trace in their travel documents. Hence, it is extremely difficult for parents to know whether their children have returned to Shenzhen. Finally, as the Hong Kong police force has stepped up action against adolescent substance abuse in rave parties, the venues of drug abuse among young people have become more diversified. Actually, some research studies showed that adolescents abused drugs in their homes, an emerging trend that deserves our attention[4].

Against this background, in his 2007 Policy Address, the Chief Executive of the Government of the Hong Kong Special Administrative Region voiced his concern about adolescent substance abuse in Hong Kong in Paragraph 86 of the Address, which states: “Hong Kong and many other advanced cities face similar social problems, among which youth drug abuse figures prominently. A lack of awareness, coupled with peer influence and curiosity, has led many young people to believe that taking psychotropic drugs is not that serious or even trendy. Drug abuse is dangerous to health as well as a criminal offence. I am deeply concerned about the problem of juvenile drug abuse because young people are the pillars of our future. We must tackle this issue with a multi-pronged approach. Otherwise, our society will definitely pay a high price in the future. To this end, I will appoint the Secretary for Justice, the incumbent Deputy Chairman of the Fight Crime Committee, to lead a high level inter-departmental task force which will make use of the existing anti-crime and anti-narcotics networks to consolidate strategies to combat juvenile drug abuse from a holistic perspective. The task force’s terms of reference covers a wide range of areas, such as preventive education and publicity, treatment and rehabilitation, law enforcement, research and external co-operation. Task force members will do their best to mobilize various government departments and the local community to tackle juvenile drug abuse”[5].

**TABLE 2**
**Type of Drug Abused in the Central Registry of Drug Abuse**

| | 1997 | 1998 | 1999 | 2000 | 2001 | 2002 | 2003 | 2004 | 2005 | 2006 |
|---|---|---|---|---|---|---|---|---|---|---|
| (1) Drug abusers with type of drugs reported | | | | | | | | | | |
| No. | 16,496 | 15,746 | 15,203 | 16,424 | 16,333 | 15,939 | 13,960 | 14,527 | 13,931 | 13,130 |
| (2) Heroin abusers | | | | | | | | | | |
| No. | 14,291 | 13,588 | 13,003 | 12,188 | 11,575 | 11,826 | 10,357 | 10,147 | 9,757 | 8,101 |
| *% of (1)* | *86.6* | *86.3* | *85.5* | *74.2* | *70.9* | *74.2* | *74.2* | *69.8* | *70.0* | *61.7* |
| (3) Psychotropic substance abusers | | | | | | | | | | |
| No. | 3,488 | 3,412 | 3,549 | 5,561 | 6,022 | 5,581 | 5,219 | 6,196 | 6,335 | 7,364 |
| *% of (1)* | *21.1* | *21.7* | *23.3* | *33.9* | *36.9* | *35.0* | *37.4* | *42.7* | *45.5* | *56.1* |
| Ketamine abusers | | | | | | | | | | |
| *% of (1)* | * | *0.0* | *0.2* | *9.8* | *16.8* | *16.9* | *14.0* | *17.8* | *15.1* | *23.2* |
| Triazolam/Midazolam/Zopiclone abusers | | | | | | | | | | |
| *% of (1)* | *5.9* | *5.7* | *6.1* | *5.6* | *5.5* | *7.8* | *11.2* | *12.1* | *14.6* | *16.9* |
| Cannabis abusers | | | | | | | | | | |
| *% of (1)* | *8.0* | *8.9* | *8.5* | *8.7* | *7.5* | *8.1* | *7.5* | *7.7* | *8.2* | *7.4* |
| MDMA (Ecstasy) abusers | | | | | | | | | | |
| *% of (1)* | *0.4* | *0.4* | *2.3* | *14.2* | *13.9* | *8.6* | *7.0* | *8.8* | *12.2* | *11.6* |
| Methylamphetamine (Ice) abusers | | | | | | | | | | |
| *% of (1)* | *5.1* | *6.0* | *6.7* | *5.9* | *5.8* | *3.8* | *4.1* | *4.4* | *5.4* | *6.5* |
| Cough medicine abusers | | | | | | | | | | |
| *% of (1)* | *2.7* | *1.8* | *1.9* | *1.9* | *1.8* | *2.4* | *3.9* | *4.5* | *5.1* | *5.7* |

*Percentage less than 0.05

How should the Hong Kong Government and community, particularly youth workers and researchers on adolescent substance abuse, respond to the Government's concern regarding tackling juvenile drug abuse? In this paper, three aspects of the issue are examined. First, ecological factors within the Hong Kong context that contribute to adolescent substance abuse are reviewed. Second, strategies and action plans based on the risk factors identified are examined. Finally, the directions that the Government of Hong Kong Special Administrative Region should and should not go regarding tackling adolescent substance abuse problems are discussed.

## The Quest for an Ecological Understanding of Adolescent Substance Abuse

At the very beginning, a proper understanding of the issue of adolescent substance abuse is in order. Although adolescent substance abuse can be understood in terms of different perspectives, the ecological perspective focusing on risk and protective factors has commonly been used to guide intervention strategies[6]. There are numerous research studies showing that risk factors at the individual level (e.g., high sensation seeking, meaninglessness), family level (e.g., growing up in nonintact families), school level (e.g., low academic achievement, poor peer relationships), and community level (e.g., growing up in deprived communities) increase the chance of drug abuse in adolescents[7,8]. On the other hand, there is also a vast literature showing that adolescents experiencing adversity do not necessarily end up as failures. Research findings showed that adolescents may adjust well despite the presence of adversity[9]. According to Hauser[10], protective factors are "key constructs in conceptualizations of resilience" that "moderate the effects of individual vulnerabilities or environmental hazards, so that a given developmental trajectory reflects more adaptation in a given domain than would be the case if protective processes were not operating" (p. 4). Based on a review of the resilience literature, Hauser outlined several categories of protective factors, including individual (e.g., healthy attribution style, self-efficacy, hope, faith), relational (e.g., supportive home environment), community (e.g., good schools and other community assets), and general (e.g., good fortune) protective factors. In a review of stress, coping, and resilience in children and youth, Smith and Carlson[11] similarly suggested that individual factors (e.g., optimism and faith), family factors (e.g., parental support and guidance), and external support systems (e.g., supportive nonparent adults) are important protective factors for children and adolescents experiencing environmental hazards. Utilizing research findings related to risk and protective processes in adolescent resilience, developers of positive youth development programs have attempted to reduce the impact of risk factors, but promote the influence of protective factors via the developed programs. Historically, the utilization of risk and protective factors has shaped the "prevention science" perspective[12]. Obviously, Hong Kong can learn much from this literature, and devise relevant policies and services to tackle the problem of adolescent substance abuse, which is consistent with the "holistic perspective" emphasis in the 2007 Policy Address.

With reference to the research findings based on the ecological perspective, there are factors at different levels that contribute to the worrying adolescent substance abuse trend in Hong Kong. On the personal level, research findings showed that several factors predispose the adolescent substance abuse problem. First, research findings have consistently showed that curiosity was a major factor leading to substance abuse among young people. Second, with material affluence and the rise of the number of nuclear families in Hong Kong, young people generally grow up without much turmoil. As such, they can be considered as growing up in a "greenhouse", where their relatively smooth upbringing would make them difficult to handle life adversities. Unfortunately, except for some isolated effort, there are no systematic life skills and positive youth development programs in the formal curriculum in Hong Kong that help adolescents to cope with life adversities[13]. Third, there are researches showing that a lack of life meaning is closely related to adolescent substance abuse. As Hong Kong has high social stress and there is a morbid emphasis on achievement[14], developing positive values in searching for life meaning may be difficult. Finally, adolescents with underachievement and nonengagement (i.e., adolescents without study and work) are also prone to substance abuse[15].

There are two factors on the interpersonal system that increase the risk of adolescent substance abuse. First, the CRDA data have consistently showed that peer influence is a strong factor contributing to adolescent substance abuse. In fact, with the rapid growth of the youth subculture and intensification of virtual communication among young people (e.g., creating blogs that are only accessible by peers, but not parents), undesirable peer influence is an important factor that may contribute to adolescent substance abuse[16]. Second, with the growing number of nuclear families (i.e., families with few children) in Hong Kong, young people have fewer opportunities to practice interpersonal skills. Hence, they are easily susceptible to the influence of negative interpersonal experiences (e.g., conflict resolution and break up in love affairs) and they take drugs to cope with negative experiences associated with interpersonal difficulties.

There are several factors on the family level that are relevant to adolescent substance abuse in Hong Kong. First, with more parents working across the border, parenting in such families is weakened. Second, with reference to the rising number of cross-border marriages (old husband and young wife syndrome), adolescent children in such families are easily influenced by parental marital problems and parenting problems. Third, with the rise of divorce rates in the past decades, parental absence (both physically and psychologically) is a prominent problem in nonintact families. There are research findings showing that adolescent developmental outcomes are worse in nonintact families than in intact families. Fourth, there are research findings showing that parental supervision in adolescent children was loose[17,18] and parents focused more on academic excellence at the expense of value development in adolescents[19]. Finally, according to the Social Development Index project undertaken by the Hong Kong Council of Social Service, there has been a substantial drop in family solidarity. From a family perspective, the problem of adolescent substance abuse should be understood in terms of dysfunctional family processes.

On the societal level, there are several indigenous factors that contribute to adolescent substance abuse in Hong Kong. First, besides substance abuse, the problems of pathological gambling[20] and internet addiction[21] have become growing problems in Hong Kong. With the growing addiction culture that tends to create the false impression that addiction is basically not bad and normal, young people's attitudes to psychotropic drugs are adversely affected. Second, with the popularity of postmodern thoughts that posit that there is no absolute standard that differentiates "right" from "wrong", young people may simply think that substance abuse is a trendy life style. Third, availability of drugs is a factor contributing to adolescent substance abuse. In particular, the light punishment for cases involving psychotropic drugs would not have deterring effects for the sellers. For example, some retailers of cough medicine abuse actually include the possible fine that they have to pay in their operating cost[4]. Fourth, with easy travel across the border, it is easy for young people to purchase and abuse drugs in the mainland with very low cost involved. In addition, the use of electronic home-return permits would make it virtually impossible for the parents to discover that their children have returned to Shenzhen. Fifth, the growing poor population creates more poor adolescents who usually have limited opportunities for personal development and they are prone to academic underachievement. Finally, growing pessimistic values and beliefs about having upward social mobility among the poor is a factor contributing to adolescent substance abuse. A summary of the risk factors contributing to adolescent substance abuse can be seen in Table 3.

**TABLE 3**
**Summary of Ecological Factors Contributing to Adolescent Substance Abuse in Hong Kong**

| Ecological System | Risk Factors |
|---|---|
| Personal | Curiosity |
| | Lack of psychosocial competencies and coping skills (growing up in a "greenhouse") |
| | Underachievement |
| | Nonengagement |
| | Hopelessness, emptiness, and lack of life meaning |
| Interpersonal | Undesirable peer influence in relation to growing emphasis of peer recognition (e.g., blogs) |
| | Few siblings in the family to practice psychosocial skills (e.g., conflict resolution) |
| School | Underachievement |
| | Undesirable after-school activities |
| Family | Cross-border working parents |
| | Cross-border marriages (old husband and young wife syndrome) |
| | Marital disruption and parental absence (both physically and psychologically) |
| | Loose parental supervision (morbid focus on academic excellence at the expense of value development) |
| | Acute decline in family solidarity |
| Societal | Growing addiction culture |
| | Postmodern youth culture |
| | Availability of drugs (light punishment for cases involving psychotropic substances); fine calculated in the cost of operating drug retailing business |
| | Availability of drugs (cross-border consumption) |
| | Pathological emphasis on achievement leading to youth demoralization and mental health problems |
| | Growing poor adolescent population |
| | Growing pessimistic values and beliefs about having upward social mobility |

## Strategies and Action Plan to Tackle the Problem

With reference to the factors that contribute to the adolescent substance abuse problem, possible strategies that can be adopted to tackle the problem are summarized in Table 4. There are several points in this table that deserve our attention. First, as there is no systematic positive youth development or drug prevention programs utilizing a curricular approach in the formal curriculum, there is a great need to consider this issue. It should be noted that without systematic coverage of related topics in the curriculum, there is no guarantee that children and adolescents get adequate inoculation against substance abuse[6]. Although the Narcotics Division conducts regular school talks, such talks are usually “one shot” in nature and many schools simply express that their students do not have the need for substance abuse prevention. There are many studies in the West showing that effective substance abuse prevention programs can reduce substance abuse behavior in the program participants[22]. As stated by Weissberg[23], successful positive youth development programs can improve the lives of millions of school children. As Hong Kong is an international city, the Hong Kong Government has to consider seriously the implementation of substance abuse prevention programs and/or positive youth development programs in a systematic and mandatory manner for children and adolescents in Hong Kong.

Second, it is obvious that there are many family factors contributing to substance abuse problems in young people. In fact, a review of both the Western and Chinese literature showed that many family factors, including systemic and dyadic parent-child factors, were related to adolescent delinquency and substance abuse[24]. Moreover, healthy family conditions are social capital that can promote healthy adolescent development and protective factors that can help adolescents face adversity. Furthermore, morbid emphasis on academic achievement and relative negligence of the holistic development of children in contemporary Hong Kong culture[19] also deserves our attention of how such cultural attributes can be changed. Third, ecological analyses in Table 3 showed that factors at different levels are associated with adolescent substance abuse. The prevention implications of this analysis are that relevant factors on different levels should be included and it is rather futile to tackle the adolescent substance abuse problem by simply changing factors at one level (e.g., increasing young people’s knowledge about drugs and changing their attitudes at the personal level). Finally, prevention work at different levels, particularly secondary prevention and primary prevention, should be stepped up.

Obviously, different time frames and priorities would be involved in formulating policies and services based on the strategies outlined in Table 4. Based on the above discussion, it is suggested that the following action plan be considered:

- There is an urgent need to re-examine the sentencing guidelines for offences related to psychotropic substances to reflect the seriousness of the related offences.
- There is an urgent need to re-examine the immigration policy for allowing children under 18 to use the home-return permits to return to China alone, particularly for cases under probation orders/care or protection orders, and/or with previous drug addiction history.
- Systematic mechanisms related to early identification of adolescents with high risk for substance abuse should be devised in the family, school, and community contexts.
- Proactive mechanisms for early identification of at-risk families (parental absence, families easily used by adolescents as venues for substance abuse) should be examined. Promotion of family competence and/or provision of alternative healthy family environment are possible intervention directions that should be explored. As there is a lack of coordinated family policies in Hong Kong, it is a prior area on which the Government should focus in the near future.

**TABLE 4**
**Strategies with Reference to the Personal, Interpersonal, School, Family, and Societal Systems**

| Ecological System | Risk Factors | Possible Strategies |
|---|---|---|
| Personal | Curiosity | Systematic drug education |
| | Lack of psychosocial competencies and coping skills (growing up in a “greenhouse”) | Systematic and holistic positive youth development programs (severely lacking in the junior secondary school years) |
| | Underachievement | Services focusing on creating success experiences for high-risk youth |
| | Nonengagement | Engagement services |
| | Hopelessness, emptiness, and lack of life meaning | Systematic and holistic positive youth development programs |
| Interpersonal | Undesirable peer influence in relation to growing emphasis of peer recognition (e.g., blogs) | Systematic and holistic positive youth development programs |
| | Few siblings in family to practice psychosocial skills | Systematic and holistic positive youth development programs |
| School | Underachievement | Creating success experiences for underachievers |
| | Undesirable after-school activities | Meaningful after-school activities |
| Family | Cross-border working parents | Parenting education |
| | Cross-border marriages (old husband and young wife syndrome) | Fine tuning of population policy; family life education |
| | Parental absence (both physically and psychologically) | Mentorship or surrogate parents; institutional care |
| | Loose parental supervision (morbid focus on academic excellence at the expense of value development) | Systematic parenting education |
| | Parental marital disruption | Mandatory parenting education for divorced cases |
| | Dropping of family solidarity | Strengthening family competence |
| Societal | Growing addiction culture (pathological gambling, internet addiction, substance abuse) | Systematic drug education; positive youth development programs |
| | Growing poor population | Mandatory parenting education for Comprehensive Social Security Assistance (CSSA) recipients |
| | Growing pessimistic values and beliefs about having upward social mobility | Specialized positive youth development program; mentorship program; subcultural changes |
| | Postmodern culture (e.g., youtube.com) | Antipostmodern culture (e.g., godtube.com) |
| | Availability of drugs (light punishment for cases involving psychotropic substances); fine calculated in the cost of operating drug retailing business | Revision of sentencing guidelines; stepping up law enforcement action |
| | Availability of drugs (cross-border consumption) | Revision of immigration policy: parental consent must be sought for children under 18 to use home-return permits to return to China alone, particularly for cases under court orders and/or with previous drug addiction history |
| | Pathological emphasis on achievement leading to youth demoralization and mental health problems | Community education; a new “cultural revolution” toward holistic development of young people; systematic positive youth development programs |

- Promotion of parenting education in the general population and specialized parenting education for parents with special needs (e.g., CSSA families and single-parent families) regarding substance abuse prevention.
- The Government should seriously consider including systematic and universal drug education and positive youth development programs in the junior secondary school years. Without such an inoculation effort, a wide range of adolescent developmental problems could emerge.
- Increase the number of social workers for schools admitting students with low academic achievement and schools in areas with a high risk for substance abuse. An increase in the number of police liaison officers in schools would also be helpful. Increase in professional manpower does make a difference in those needy schools.
- It is noteworthy that research on psychotropic substance abuse is severely lacking in the international and local contexts. Research findings are vital as far as evidence-based services and policy formulation are concerned. As such, systematic research studies should be carried out.

## Directions where Hong Kong Should and Should Not Go

In devising policies for tackling adolescent substance abuse, the Government should carefully note that existing research findings have implications for the directions where Hong Kong should go as well as should not go. As shown in Table 5, the following directions should be avoided: (a) research findings have showed that scary tactics do not work; (b) blaming adolescents for their weaknesses alone do not work; (c) understanding the problem from a single perspective does not help; (d) formulation of policies and services with good intention only is futile; (e) supply reduction cannot totally solve the problem because demand still exists; (f) formulation of policies based on adults only will miss the viewpoints of young people; (g) policies devised by the Government alone erode the sense of ownership in different stakeholders, particularly in adolescents; (h) resources used mainly on treatment cannot help to tackle the problem of adolescent substance abuse.

**TABLE 5**
**Directions Hong Kong Should Go (and Should Not Go) as Far as Strategies for Tackling Adolescent Substance Abuse are Concerned**

| Should Not Go | Should Go |
|---|---|
| Scare adolescents | Understand adolescents |
| Blaming adolescents for their weaknesses | Understanding ecological factors leading to the problem |
| Understand the problem from a single perspective (e.g., personal problems) | Ecological understanding, particularly family-based intervention, is important |
| Formulation of services and policies with goodwill only | Evidence-based and research-driven services and policies |
| Supply reduction strategies only | Supply reduction and demand reduction strategies |
| Services and policies involving adults only | Services and policies with the involvement of adults and adolescents |
| Policies devised by the Government alone | Policy making involving stakeholders in different sectors |
| Resources mainly devoted to treatment | Resources devoted to primary, secondary, and tertiary prevention |

In contrast, the following directions should be considered by Hong Kong: (a) while pointing out that adolescent substance abuse is a “problem”, understanding adolescents is equally important; (b) it is important to understand how different ecological factors lead to adolescent substance abuse; (c) ecological understanding, particularly family-based intervention, is of paramount importance; (d) evidence-based and research-driven services and policies should be desired; (e) supply reduction and demand reduction are equally important; (f) involvement of adolescents in the process of formulating services and policies is important; (g) policy making involving stakeholders in different sectors is needed; (h) resources devoted to primary, secondary, and tertiary prevention are equally important.

## CONCLUSIONS

In conclusion, this paper argues for an ecological understanding of the phenomenon of adolescent substance abuse in Hong Kong so that a more holistic understanding can be achieved. This approach is consistent with the spirit of "holistic perspective" as stated in the 2007 Policy Address of the Chief Executive of the Government of the Hong Kong Special Administrative Region. I earnestly hope that through this paper, further discussion incorporating the concepts of risk factors, protective factors, at-risk families, ecological perspective, primary prevention, secondary prevention, tertiary prevention, positive youth development, and holistic adolescent development in exploring a more long-term and coordinated approach in tackling adolescent substance abuse in Hong Kong will be carried out.

## ACKNOWLEDGMENTS

The preparation for this paper was financially supported by the Wofoo Foundation Limited.
